# A Case of Overlapping Autoimmune Syndrome

**DOI:** 10.7759/cureus.59714

**Published:** 2024-05-06

**Authors:** Moses O Evbuomwan, Toyin Ingram, Nafisa Saleem

**Affiliations:** 1 Radiation Oncology, University of Iowa Hospitals & Clinics, Iowa, USA; 2 Internal Medicine, Cape Fear Valley Health, Fayetteville, USA

**Keywords:** eosinophilic granulomatosis with polyangiitis, acute polymyositis, polymyositis, autoimmune disorder, interstial lung disease, systemic lupus erythematosus disease, anti-synthetase syndrome, : rheumatoid arthritis, systemic sclerosis, mixed connective tissue disease

## Abstract

Overlapping autoimmune disorders are used to describe the coexistence of more than one autoimmune disease in the same patient. Mixed connective tissue disease (MCTD) and anti-synthetase syndrome (ASS) are autoimmune diseases that manifest with pulmonary involvement, presenting as persistent dyspnea. The coexistence of both conditions in the same patient is extremely rare. We herein report a case of a 44-year-old female who was diagnosed with MCTD with features of ASS (anti-Jo-1 antibody) in the setting of rheumatoid arthritis (anti-cyclic citrullinated peptide (anti-CCP) antibody), which shows temporary breathing improvement following treatment with corticosteroid and mycophenolate mofetil. However, after the completion of mycophenolate mofetil, she was found to be anti-Jo-1 antibody negative and anti-CCP antibody positive. Our case emphasizes the need to recognize overlapping autoimmune conditions in patients with complex clinical features and presentations with the immediate application of a comprehensive diagnostic approach and tailored treatment strategies. Early diagnosis and aggressive treatment are crucial for achieving remission and preventing organ damage.

## Introduction

Overlap autoimmune syndrome is a term used to describe patients who have two coincident autoimmune diseases [1]. It describes the coexistence of more than one autoimmune disease in the same patient [2]. Few case reports have described overlap autoimmune syndrome, including the diagnosis of eosinophilic granulomatosis with polyangiitis and mixed connective tissue disease (MCTD) in a 27-year-old man [2].

MCTD, also known as Sharp syndrome, is a rare systemic autoimmune disorder with an unknown cause [3]. MCTD presents with overlapping features of at least two connective tissue diseases, such as systemic lupus erythematosus (SLE), systemic sclerosis (scleroderma), inflammatory myopathy (polymyositis (PM) or dermatomyositis (DM)), and rheumatoid arthritis (RA), along with the presence of antibodies directed against U1-ribonucleoprotein (RNP) [4]. In the United States, the disease is estimated to be less than 50,000, although the exact incidence is unknown [3]. Nonetheless, studies have shown that the disease is more common in females than males and affects all races with similar clinical manifestations, irrespective of ethnic group [5,6]. Similarly, anti-synthetase syndrome (ASS), also known as anti-Jo-1 syndrome, is a chronic autoimmune condition [7]. ASS is associated with the presence of antibodies directed against one of the several aminoacyl-transfer RNA (tRNA) synthetases, the most commonly known being the anti-Jo-1 antibody (anti-histidyl-tRNA synthetase) [8]. Some of the clinical manifestations associated with ASS include interstitial lung disease (ILD), inflammatory myopathy (PM or DM), inflammatory arthritis, and Raynaud’s phenomenon [7,9]. Also, ASS prevalence in the United States is estimated to be less than 50,000 [5].

Here, we describe a complex case of a 44-year-old female with a past medical history of asthma and RA (anti-cyclic citrullinated peptide (anti-CCP) antibody positive) who was diagnosed with MCTD with features of ASS (anti-Jo-1 antibody positive result: 3.9 (0-0.9 AI)). Her dominant ILD showed only temporary breathing improvement following treatment with corticosteroids and mycophenolate mofetil. She was subsequently found to be anti-Jo-1 antibody negative (<20 units) but retained her anti-CCP antibody positive status nine months post-hospitalization after completion of mycophenolate mofetil.

## Case presentation

A 44-year-old female with a past medical history of essential hypertension, asthma, and RA presented to the ER with concerns of worsening exertional dyspnea for a month. Of note, she had recently been diagnosed with RA (anti-CCP antibody, 250 units; reference range: 0-19 units) and had completed two months of methotrexate therapy (10 mg weekly).

**Figure 1:**
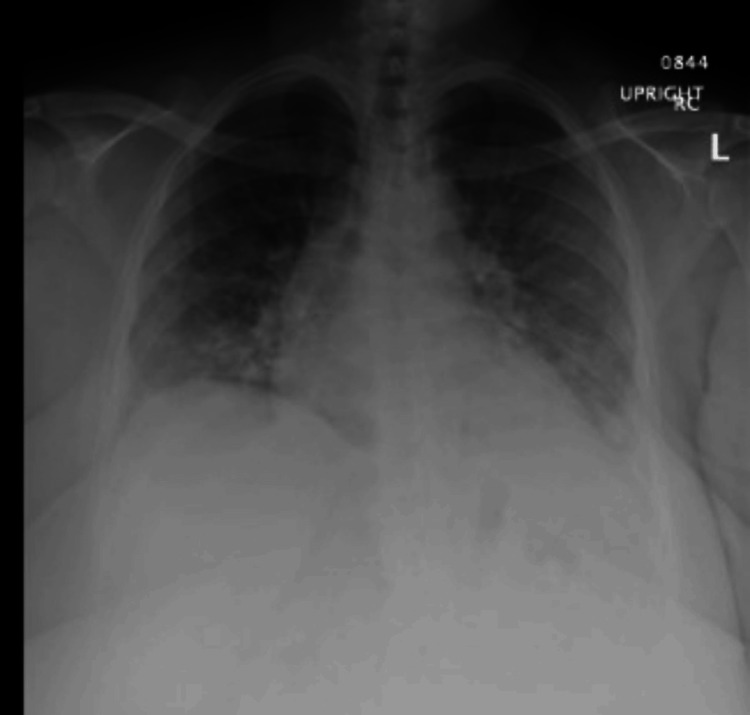
A chest X-ray obtained upon admission revealed emerging bilateral perihilar infrahilar patchy infiltrates, predominantly airspace in nature, extending inferiorly

She presented to the ER with concerns about dyspnea. On arrival, her vital signs were as follows: blood pressure of 162/96, heart rate of 108, respiratory rate of 24, temperature of 37.2 °C (99 °F), and saturation of 96% on room air. She was subsequently diagnosed with community-acquired pneumonia and discharged on azithromycin. A month later, she presented to the ER with complaints of a productive cough and blood-tinged sputum. A chest X-ray revealed bilateral infiltrates (Figure 1), and she was again discharged from the ER on azithromycin for the second time. However, her condition did not improve, and she was treated with doxycycline and amoxicillin for one week as well as 10 days of prednisone by both her urgent care physician and her primary care physician, respectively, after her two recent ER visits.

**Figure 2:**
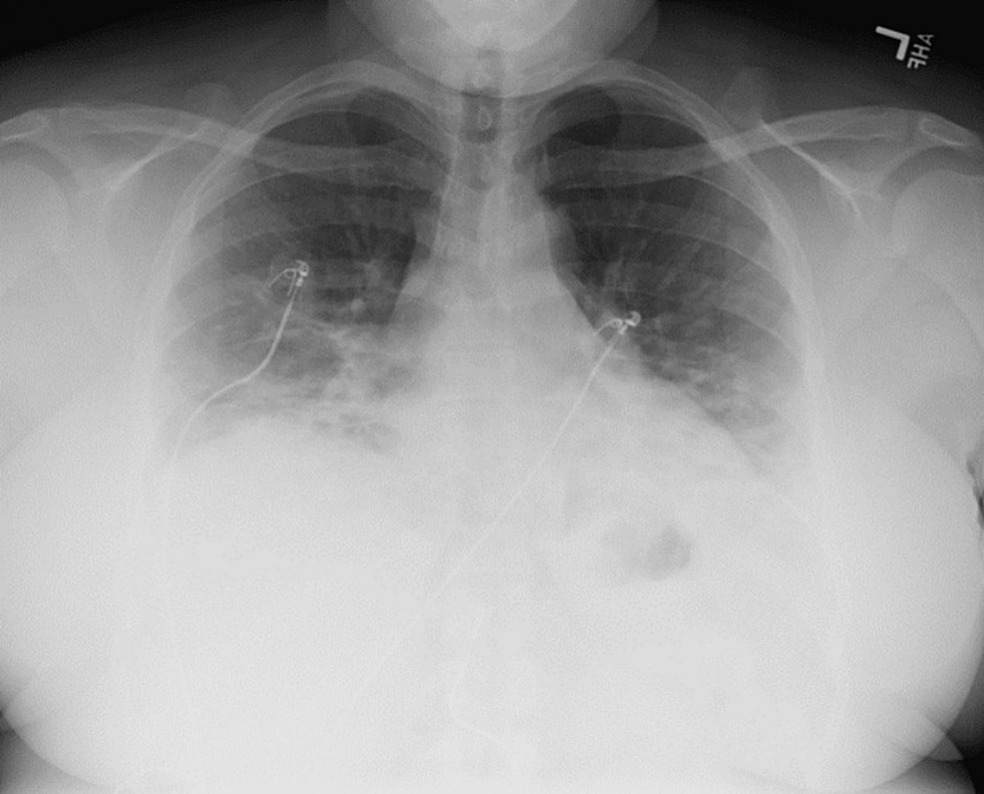
A chest X-ray taken five days after the initial X-ray revealed extensive bilateral airspace disease with a differential of pulmonary edema versus multifocal pneumonia

Then, five days prior to her current presentation, she returned to the ER for the third time with complaints of subjective fever, chills, myalgias, and increased difficulty breathing. A repeated chest X-ray at this time showed extensive bilateral airspace disease associated with pulmonary edema, raising concern for multifocal pneumonia (Figure 2).

The patient was noted to be mildly hypoxic, requiring 2 liters of oxygen via a nasal cannula. She was admitted and treated with ceftriaxone, doxycycline, and prednisone, with an improvement in her respiratory status saturating at 92% on room air. She was subsequently discharged home on Avelox (moxifloxacin hydrochloride) therapy along with 40 mg of prednisone for a total of five days for presumed community-acquired pneumonia and prescribed long-acting bronchodilators at the time of discharge. In one month, the patient had received multiple courses of antibiotic therapy without returning to her prior respiratory baseline status.

**Figure 3:**
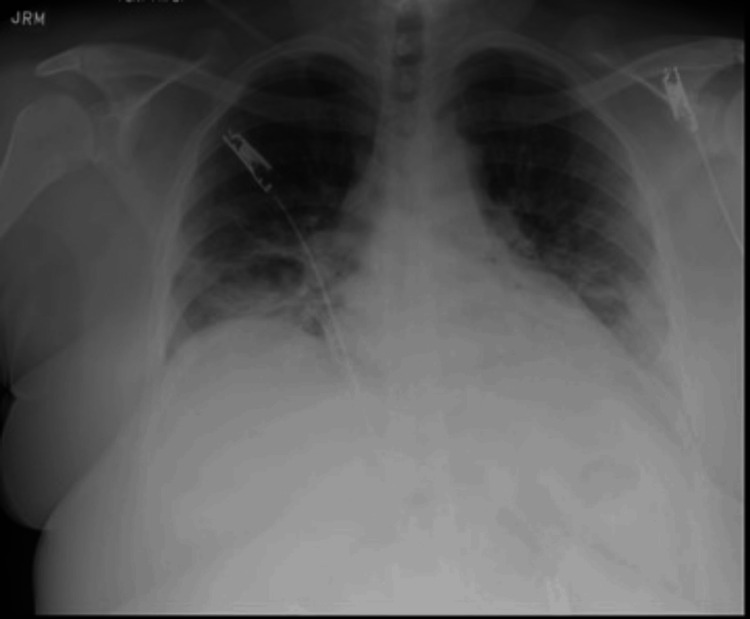
A chest X-ray taken 10 days after the initial presentation revealed residual basilar airspace opacity, as compared to Figure 2

**Figure 4:**
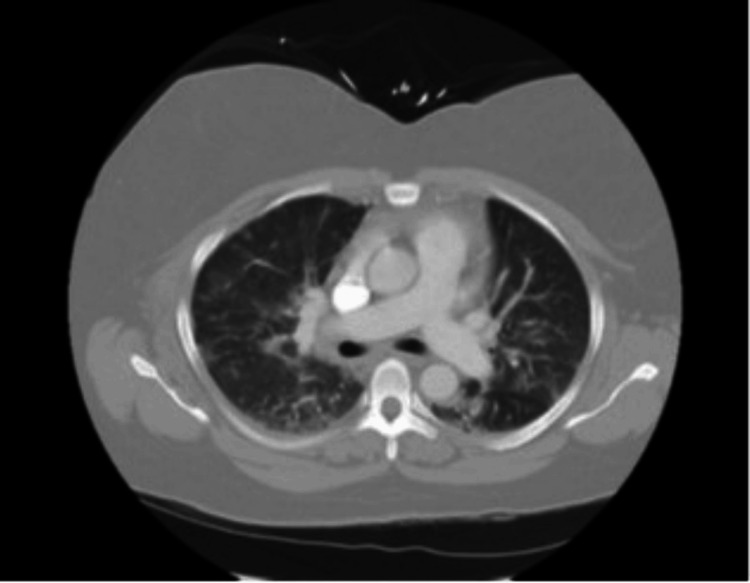
A CT chest with and without contrast conducted upon admission revealed the presence of lung infiltrates

Despite all the antimicrobial therapy, the patient continued to report worsening dyspnea, which prompted her current and fourth ER visit. During this current presentation, she reported fatigue, congestion, cough, nausea, and weakness. Her pulmonary physical examination revealed clear upper lung fields, crackles at the bases that were not present on the prior physical examination, as well as coughing throughout the examination. Her oxygen saturations were found to be in the mid-80s on room air, and no arterial blood gases were completed. She was also found to be afebrile and hypoxic, saturating 90% of the room air. She was subsequently placed on supplemental oxygen via a nasal cannula. The patient’s complete blood count was significant for leukocytosis of 15.2K, erythrocyte sedimentation rate of 107, and C-reactive protein of 65. Her complete metabolic panel assessing her kidney and liver function was unremarkable. Her nasal swab was tested for methicillin-resistant *Staphylococcus aureus*, the respiratory pathogen panel for SARS-CoV-2, respiratory syncytial virus, and flu, and all came back negative. However, her respiratory culture and stain were positive for rare gram-positive cocci, whereas results for Aspergillus galactomannan antigen, Fungitell, aldolase, HIV, creatine kinase, ferritin, and Histoplasma galactomannan antigen were all negative. Her chest X-ray done in the ER during this visit revealed residual basilar airspace opacity (Figure 3), and her CT with and without contrast of her chest did not reveal pulmonary embolism but demonstrated extensive interstitial and airspace infiltrates (Figure 4). Her fungal and viral infection workups were all negative.

Given that the patient had endured multiple courses of antibiotics with no improvement, there was a reasonable suspicion that her lung disease was not of an infectious etiology. An infectious disease physician who was consulted recommended all antimicrobial agents be discontinued. Pulmonology was consulted, and bronchoscopy was performed under anesthesia. A total of 15 mL of clear red fresh fluid was collected and sent to the lab, where one thin prep slide was prepared. Results yielded benign bronchial cells with lymphocytes and macrophages from her right lung lower lobe. Her bronchoscopy with tracheobronchial biopsies revealed no evidence of an acute infectious process, granulomatous inflammation, or malignancy. However, since the cell count was noted to be predominantly lymphocytes, there was a suspicion for autoimmune pathology. An antinuclear antibody panel was obtained, and the results were positive for anti-RNP antibody (1.6 (0-0.9 AI)), anti-Jo-1 antibody (3.9 (0-0.9 AI)), and anti-centromere antibody (3.0 (0-0.9 AI)). Her prognosis and laboratory findings were discussed over the phone with her outpatient rheumatologist, who suspected the diagnosis of MCTD with features of ASS in the setting of RA. The rheumatologist recommended initiation of any of the available therapeutic options, including rituximab (first line, 1 g infusion twice 14 days apart) or azathioprine (50 mg BID; however, azathioprine required thiopurine methyltransferase enzyme level testing before initiation because the enzyme breaks down a class of drugs called thiopurines) or mycophenolate mofetil (500 mg BID for one week, then 1 g BID thereafter). We opted to manage her with 125 mg IV solumedrol BID for three days, followed by 40 mg of prednisone daily and 500 mg IV mycophenolate mofetil BID for seven days, followed by 1 g mycophenolate mofetil BID for four weeks. Unfortunately, given the lack of in-house rheumatologic services at the time and the lack of familiarity with the administration of rituximab by the team, attempts were made to transfer the patient to a nearby tertiary institution for rheumatology services. She was accepted and placed on a waiting list for a bed for several days. In the meantime, 500 mg IV mycophenolate mofetil BID for seven days was started, with minimal side effects of nausea and abdominal cramping reported. Her reported side effects were well managed with oral promethazine. She tolerated room air at rest, although she now required 3 liters of oxygen via nasal cannula for ambulation. Unfortunately, after a five-day wait for a bed at the tertiary institution, she requested to be discharged. She was stable for discharge and sent home on 1 g mycophenolate mofetil BID for a month, prednisone 20 mg daily, and a 3-liter home oxygen prescription, as well as instructions to follow-up with her primary care physician, rheumatologist, and pulmonologist.

During her post-hospitalization follow-up at the pulmonology office four weeks later, the patient reported unabated shortness of breath and dyspnea on exertion. She had not returned to her baseline oxygen requirement and had remained on home oxygen for ambulation. Two months post-discharge, she was started on a rituximab 20 mg infusion every two weeks. Then, roughly nine months after she was last seen by our multidisciplinary team, she had extensive laboratory work completed at another tertiary institution, about 90 miles north of ours. Her extensive autoimmune panel workup revealed negative results for the following markers: Myo Marker 3, MAYO, anti-Jo-1 antibody, anti-PL7 antibody, anti-PL12 antibody, anti-OJ antibody, anti-EJ antibody, anti-SRP antibody, anti-MI-2 antibody, anti-TIF-1 gamma antibody, anti-MDA-5 antibody (CADM-140), anti-NXP-2 antibody (p140), anti-PM/SCL-100 antibody, anti-KU antibody, anti-SSA 52-kD antibody IgG, anti-U1 RNP antibody, anti-U2 snRNP antibody, and anti-U3 RNP (Fibrillarin) antibody.

**Figure 5:**
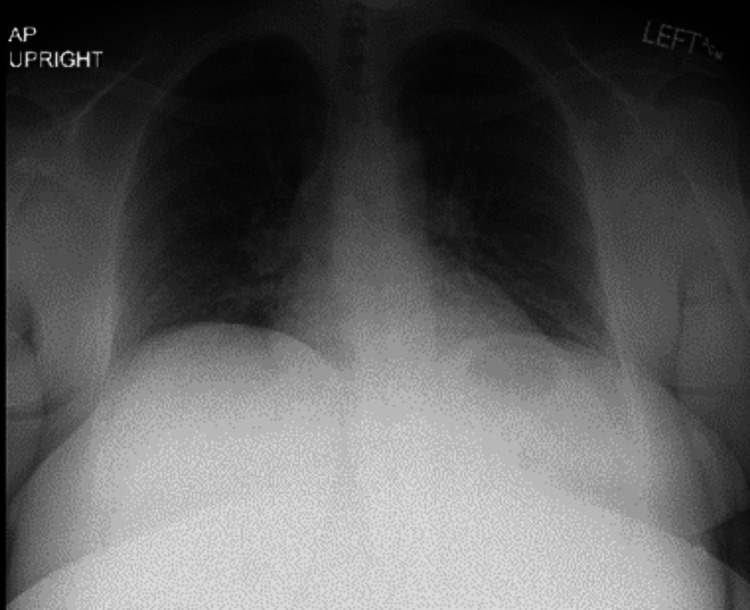
A chest X-ray conducted nearly 1.5 years later, depicting substantial improvement in lung condition

During a recent pulmonologist follow-up a year and a half after the initial presentation, she remained on a rituximab 20 mg infusion every two weeks. Also, she had already completed pulmonary rehabilitation and was off oxygen for ambulation without experiencing shortness of breath. A repeated chest X-ray revealed a partially limited evaluation of the segmental and subsegmental pulmonary arteries and moderate scattered opacities at the lung bases related to scarring or fibrosis with associated cylindrical bronchiectasis (Figure 5). No pulmonary embolism was visualized within the pulmonary arteries.

As of the time of writing this report, the patient has been successfully weaned off home oxygen for ambulation and was scheduled to see her pulmonologist and rheumatologist in three months.

## Discussion

We demonstrate a case of a patient with an overlapping autoimmune syndrome, namely MCTD and ASS. In general, the term overlapping autoimmune syndrome is used to describe the coexistence of more than one autoimmune disease in the same patient, as seen in this case [2]. MCTD is also known as an overlap syndrome, which combines features of SLE, Sjogren’s syndrome, systemic sclerosis (scleroderma), inflammatory myopathy (PM or DM), and RA, as well as positivity for anti-U1 RNP antibody [2,10]. Similarly, ASS is an autoimmune condition that presents with features of ILD, arthritis, myositis, and Raynaud’s phenomenon. The diagnostic criteria for ASS, as summarized by both Connors et al. and Solomon et al., require the presence of an anti-aminoacyl tRNA synthetase antibody plus clinical features such as ILD and arthritis, which were present in our patient [11-13].

Our patient had persistent dyspnea that was misdiagnosed because of the complex clinical features and presentation. Given her new diagnosis of RA and the recent completion of two months of methotrexate therapy (10 mg weekly) for a month prior to her first ER visit, dyspnea could have exacerbated her underlying lung disease. Once diagnosed with the overlapping autoimmune syndromes of MCTD and ASS, first-line therapy with corticosteroids and second-line therapy with mycophenolate mofetil were given to the patient [11] and the side effects she experienced were well managed with oral promethazine. Early and aggressive treatment with corticosteroids (prednisone) is especially important [13]. Also, our patient would have benefited from early rheumatologist service, especially with the start of rituximab, which has been shown to be effective for ASS symptoms, especially for patients with anti-Jo-1 antibodies [13]. Unfortunately, our institution did not have an in-house rheumatological service at that time, and we did not have experience administering rituximab as hospitalists. Also, our oncology team was reluctant to administer rituximab to a non-cancer patient.

Given the delay in diagnosis, the patient returned with worsening respiratory status - shortness of breath with minimal exertion - and required 2-3 liters of oxygen for ambulation. While she tested positive for anti-Jo-1 antibody before the initiation of treatment at our institution, she tested negative for anti-Jo-1 antibody nine months later at another nearby tertiary institution. Extensive laboratory workups involving anti-synthetase antibodies (anti-Jo-1, anti-PL7, anti-PL12, anti-OJ, anti-EJ, and anti-SC), myositis-specific antibodies (anti-SRP, anti-MDA-5, and anti-Mi-2), and myositis-associated antibodies (anti-PM-SCL, anti-KU, and anti-U2 snRNP) performed were negative. The reason for these discrepancies is unknown to us and could not be explained. Despite the non-detection of ASS-specific antibodies, the patient reports having dyspnea and using 2-3 liters of oxygen for ambulation. One of the possible reasons for persistent dyspnea is fibrosis of her lung, as ILD is more prevalent in ASS compared to PM or DM, which is also seen in MCTD.

Lastly, overlap syndrome, such as MCTD and ASS, is a subset of overlapping autoimmune diseases. In this case, we discussed the challenges involved in diagnosing patients with overlapping autoimmune diseases and the importance of early diagnosis and adequate treatment. We believe that the early administration of rituximab in combination with mycophenolate mofetil and corticosteroid would have provided a fast and rapid resolution of the disease, in line with published literature [14]. The subsequent administration of rituximab to the already-completed mycophenolate mofetil and corticosteroid in addition to pulmonary rehabilitation helped wean off the patient from home oxygen use. Due to the overlap in the phenotypic presentation of these diseases, it is important to use a multidisciplinary approach in diagnosing and managing patients with overlap syndrome.

## Conclusions

The present case is consistent with a diagnosis of overlapping autoimmune syndrome, MCTD, and ASS (anti-Jo-1 antibody) in the setting of RA (anti-CCP antibody). Awareness of the present case, as well as other similar cases, is essential for early diagnosis and comprehensive multidisciplinary management of the patients because of the overlapping phenotypic presentation of these diseases. The history of persistent and unrelieved dyspnea should clue clinicians to the diagnosis of overlapping autoimmune syndromes, especially MCTD with pulmonary and extrapulmonary involvement, to trigger early administration of combined immunosuppressive therapy with corticosteroids, mycophenolate mofetil, and rituximab.
